# Magnetophoretic sorting of microdroplets with different microalgal cell densities for rapid isolation of fast growing strains

**DOI:** 10.1038/s41598-017-10764-6

**Published:** 2017-09-04

**Authors:** Young Joon Sung, Jaoon Young Hwan Kim, Hong Il Choi, Ho Seok Kwak, Sang Jun Sim

**Affiliations:** 1 Department of Chemical and Biological Engineering, Korea University, Seoul, 136-713 Republic of Korea; 2 Convergence Research Division, National Marine Biodiversity Institute of Korea, Jangsan-ro 101beon-gil 75, Janghang-eup, Seocheon-gun, Chungcheongnam-do, 33662 Republic of Korea; 3 Department of Food Engineering, Dongyang Mirae University, 445, Gyeongin-ro, Guro-gu, Seoul 08221 Republic of Korea

## Abstract

Microalgae – unicellular photosynthetic organisms – have received increasing attention for their ability to biologically convert CO_2_ into valuable products. The commercial use of microalgae requires screening strains to improve the biomass productivity to achieve a high-throughput. Here, we developed a microfluidic method that uses a magnetic field to separate the microdroplets containing different concentrations of microalgal cells. The separation efficiency is maximized using the following parameters that influence the amount of lateral displacement of the microdroplets: magnetic nanoparticle concentration, flow rate of droplets, *x*- and *y*-axis location of the magnet, and diameter of the droplets. Consequently, 91.90% of empty, 87.12% of low-, and 90.66% of high-density droplets could be separated into different outlets through simple manipulation of the magnetic field in the microfluidic device. These results indicate that cell density-based separation of microdroplets using a magnetic force can provide a promising platform to isolate microalgal species with a high growth performance.

## Introduction

Global energy demand has steadily increased as the world population increases and living standards improve. Since the Industrial Revolution, fossil fuels with a large energy density have been used to meet energy demands. However, burning fossil fuels results in emissions of massive amounts of carbon dioxide (CO_2_) into the atmosphere. The increased level of CO_2_ in the atmosphere has great potential to contribute to global warming and climate change, resulting in serious environmental problems such as deforestation^1^, extreme weather events^2^, drought^3^, and sea-level rise^4^. Accordingly, carbon capture and sequestration (CCS) has emerged as a promising technology to mitigate the risk of the global warming. From an economic perspective, there have been many attempts to convert CO_2_ into various high-value products using photosynthetic microorganisms, including microalgae and cyanobacteria^5–7^.

Microalgae – unicellular species that are present in freshwater and marine systems – engage in photosynthesis using water, solar energy, and CO_2_ to produce organic compounds and oxygen (O_2_). In general, microalgae have many advantages compared to terrestrial plants, including a fast growth rate^8^, rapid biomass accumulation^9^, high photosynthetic efficiency^10^, and non-competition with agricultural land^11^. Therefore, we can utilize microalgae as the most promising renewable resources to convert CO_2_ into the valuable products^12–15^. However, to commercialize microalgae-based CO_2_ capture processes, it is necessary to isolate microalgal species with a fast growth rate to increase biomass productivity^16, 17^. Normally, cell growth is monitored via optical density in a microplate reader, and the growth performance of the microalgal cells is evaluated to quickly screen microalgae with a high biomass productivity. However, conventional approaches are time-consuming and labor-intensive when isolating a specific strain from about 300,000 microalgal species that have greater diversity than that of land plants^18^.

To address the limitations of such conventional approaches, microdroplet-based microfluidic systems have been employed to conduct high-throughput chemical and biological analyses^19–21^. Microdroplets generated by two immiscible phases, such as water and oil, can act as an individual microreactor. A single microdroplet with a diameter of a few microns has a high surface to volume ratio, so reactions can be carried out very quickly due to the short diffusion distance and rapid transfer of the heat and mass^22^. In addition, droplet-based microfluidics can be utilized to conduct parallel, high-throughput experiments because microdroplets can be generated at frequency of up to 10 kHz^23, 24^. Based on these advantages, it is possible to utilize microdroplets as a bioreactor to simultaneously cultivate different microalgal species^25–27^. Due to different growth rates and biomass productivities of various microalgal species, cell densities in microdroplets become different during cell cultivation. Therefore, efficient sorting technology to separate microdroplets with different cell densities can be a promising solution to quickly isolate fast-growing microalgal strains. Several studies have been carried out on cell density-based separation of microdroplets using chlorophyll fluorescence^28^ or alginate beads using a standing surface acoustic wave (SSAW)^29^ in microfluidic devices. However, these microfluidic approaches require a complex device design and a fabrication process to integrate the electrodes, piezoelectric substrates, and additional equipment, including a signal generator, amplifier, and DC power supply^28–30^.

Here, we present a novel microfluidic approach that uses a magnetic field to separate microdroplets encapsulating a different number of microalgal cells. The magnetic droplet manipulation method has the advantages of being simple and easy to manipulate over other active droplet-manipulation systems using optical^28^, acoustic^29^, and electric forces^30^. Based on these advantages, Zhang *et al*. generated monodisperse superparamagnetic droplets and manipulated droplets by changing the magnet positions and regulating the number of magnetic nanoparticles in droplets^31^. Jo *et al*. used magnetophoretic sorting system to distinguish the single cell encapsulated droplets which have a reduced number of magnetic nanoparticles that can be applied to single cell analysis^32^. To utilize the magnetophoretic sorting system as cell screening technology, we used microdroplets containing the same concentration of 20 nm iron oxide magnetic nanoparticles coated with a biocompatible polymer, dextran^33^, which are superparamagnetic and have no magnetic memory^31^. From Newton’s second law of motion, the net force acting on the magnetic microdroplets causes acceleration and lateral displacements in the *y*-axis, which depends on the mass of the microdroplets or cell density in the microdroplets. Based on a theoretical analysis, the separation efficiency can be maximized by investigating the amount of lateral displacement of the microdroplets according to various parameters, including the concentration of magnetic nanoparticles, flow rate of the droplets, *x*- and *y*-axis location of the magnet, and diameter of the droplets. In addition, stepwise increases in the expansion angle were introduced in the microchannel to separate three kinds of magnetic microdroplets with different cell densities. This microfluidic platform can be used to separate empty, low-, and high-density microdroplets with an efficiency higher than 90%.

## Results

### Theoretical analysis

In general, magnetic iron oxide nanoparticles with a diameter of sub-30 nm, such as magnetite (Fe_3_O_4_) or maghemite (γ-Fe_2_O_3_), are considered to have superparamagnetic properties^33^. In this study, we used dextran-coated magnetic nanoparticles with a diameter of 20 nm to conduct the density-based separation of magnetic microdroplets that exhibit magnetic properties when an inhomogeneous magnetic field is applied. If a magnetic field is not applied, there is no magnetism in the nanoparticles due to no magnetic memory^31^. The interfacial adhesive force is strong enough to move microdroplets containing magnetic nanoparticles and microalgal cells toward the maximum point of the magnetic field gradient (near the magnet). The magnetic force acting on the magnetic microdroplets can be expressed as the sum of the magnetic force on each magnetic nanoparticle (equation (1))^31^.

1 $${F}_{mag}=\frac{N\cdot {\rm{\Delta }}\chi \cdot {V}_{p}}{{\mu }_{0}}\cdot B\cdot (\nabla B)$$

where *N* is the number of magnetic nanoparticles in the microdroplets (dimensionless), Δ*χ* is the difference in the magnetic susceptibility between the medium and magnetic nanoparticles (10^−4^, dimensionless), *V*_*p*_ is the volume of a magnetic nanoparticle (m^3^), *µ*_0_ is the vacuum permeability (4π × 10^−7^, H m^−1^), *B* and ∇*B* are the magnetic flux density (T) and the gradient of the magnetic flux density (T m^−1^), respectively. From equation (1), the total magnetic force increases with an increase in the magnetic flux and its density, which can be determined by the distance between the magnetic nanoparticles and the magnet. When magnetic microdroplets are deflected from laminar flow due to the magnetic force, the Stokes’ drag force acts in the opposite *y*-axis direction of the magnetic force.

2 $${F}_{drag}=6\pi \cdot \eta \cdot r\cdot {v}_{y}$$

where *η* is the viscosity of the FC-40 oil (kg m^−1^ s^−1^), *r* is the radius of a single microdroplet (m), and *v*_*y*_ is the *y* component of droplet velocity (m s^−1^). This force is induced in stokes regime which have small Reynolds number (Re < 1) due to the microscale channel in microfluidics. When the droplet flows in a microchannel under laminar flow conditions (stokes regime), we can consider the droplet as a single uniform entity regardless of the number of cells in droplet or the viscosity of droplet. The net force on the magnetic microdroplets can be expressed by the magnetic and drag forces. We can substitute equation (1) for the force of the magnet and equation (2) for the drag force to obtain equation (3).

3 $${F}_{net}={F}_{mag}-{F}_{drag}=\frac{N\cdot {\rm{\Delta }}\chi \cdot {V}_{p}}{{\mu }_{0}}\cdot B\cdot (\nabla B)-6\pi \cdot \eta \cdot r\cdot {v}_{y}$$

From Newton’s second law of motion, the net force of the microdroplet can be expressed by multiplying the mass and the acceleration of the microdroplets. Thus, when the droplet initially goes through the magnetic field, there is difference in the acceleration of the droplets due to their different densities. At this moment, the droplets are attracted toward the magnet because the magnetic force is main driving force to *y*-direction movement of droplets that causes the drag force against their moving direction. We obtain the following expression (equation (4)).

4 $${F}_{net}={m}_{drop}\cdot \frac{d{v}_{y}}{dt}=\frac{N\cdot {\rm{\Delta }}\chi \cdot {V}_{p}}{{\mu }_{0}}\cdot B\cdot (\nabla B)-6\pi \cdot \eta \cdot r\cdot {v}_{y}$$

We solve the differential equation (4) assuming an initial velocity of zero (*v*_*y*_ = 0) to find the *y* component of the droplet velocity *v*_*y*_, which is given by equation (5).

5 $${v}_{y}(t)=\frac{N\cdot {\rm{\Delta }}\chi \cdot {V}_{p}\cdot B\cdot (\nabla B)}{6\pi \cdot \eta \cdot r\cdot {\mu }_{0}}\cdot (1-{e}^{-\frac{6\pi \cdot \eta \cdot r}{{m}_{drop}}\cdot t})$$

To obtain the *y* component of the lateral displacement *L*_*y*_ *(t)*, we integrate equation (5) from the initial time, zero, to its final time, *t* (equation (6)).

6 $${L}_{y}(t)={\int }_{0}^{t}{v}_{y}(t)dt=\frac{N\cdot {\rm{\Delta }}\chi \cdot {V}_{p}\cdot B\cdot (\nabla B)}{6\pi \cdot \eta \cdot r\cdot {\mu }_{0}}\cdot (t+\frac{{m}_{drop}}{6\pi \cdot \eta \cdot r}\cdot ({e}^{-\frac{6\pi \cdot \eta \cdot r}{{m}_{drop}}\cdot t}-1))$$

**Figure 1:**
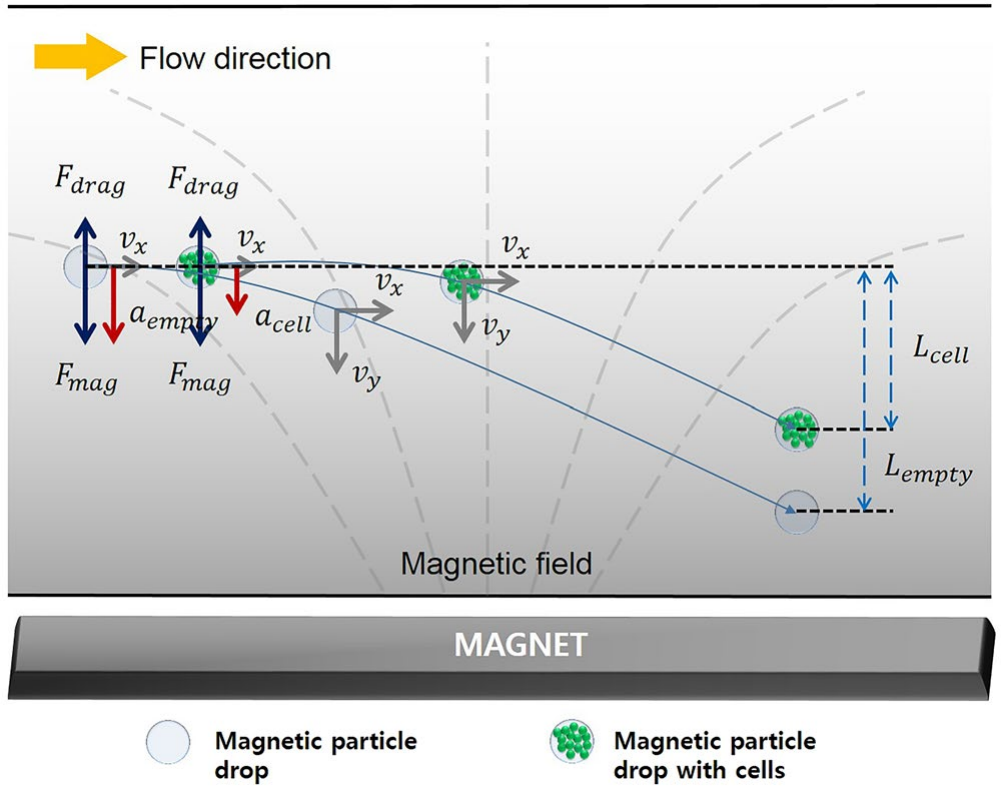
Schematic showing the separation of magnetic microdroplets with different cell densities. All magnetic droplets are attracted by the same magnetic force because they contain the same number of magnetic nanoparticles. There are differences in the number of encapsulated cells that can make different cell densities in the magnetic droplets. The net force on the magnetic microdroplet can be expressed by the difference in the magnetic and drag force. From Newton’s second law of motion, the mass of the microdroplet can determine the lateral displacement of the droplet due to the difference in the acceleration. Therefore, it is possible to separate the magnetic microdroplets with different cell densities using a magnetic field.

According to equation (6), the *y* component of the lateral displacement of the droplet can be determined by the mass of the microdroplet (*m*_*drop*_). Therefore, microdroplets containing a different number of cells show differences in the lateral displacement in the *y*-axis direction and can be separated (Fig. 1). From equation (6), it is possible to increase the difference in the lateral displacement of the droplets by regulating various parameters, such as the time duration on the magnetic field, magnetic flux density, and mass of the microdroplets.

### Design and structures of microfluidic devices used to separate the microdroplets

**Figure 2:**
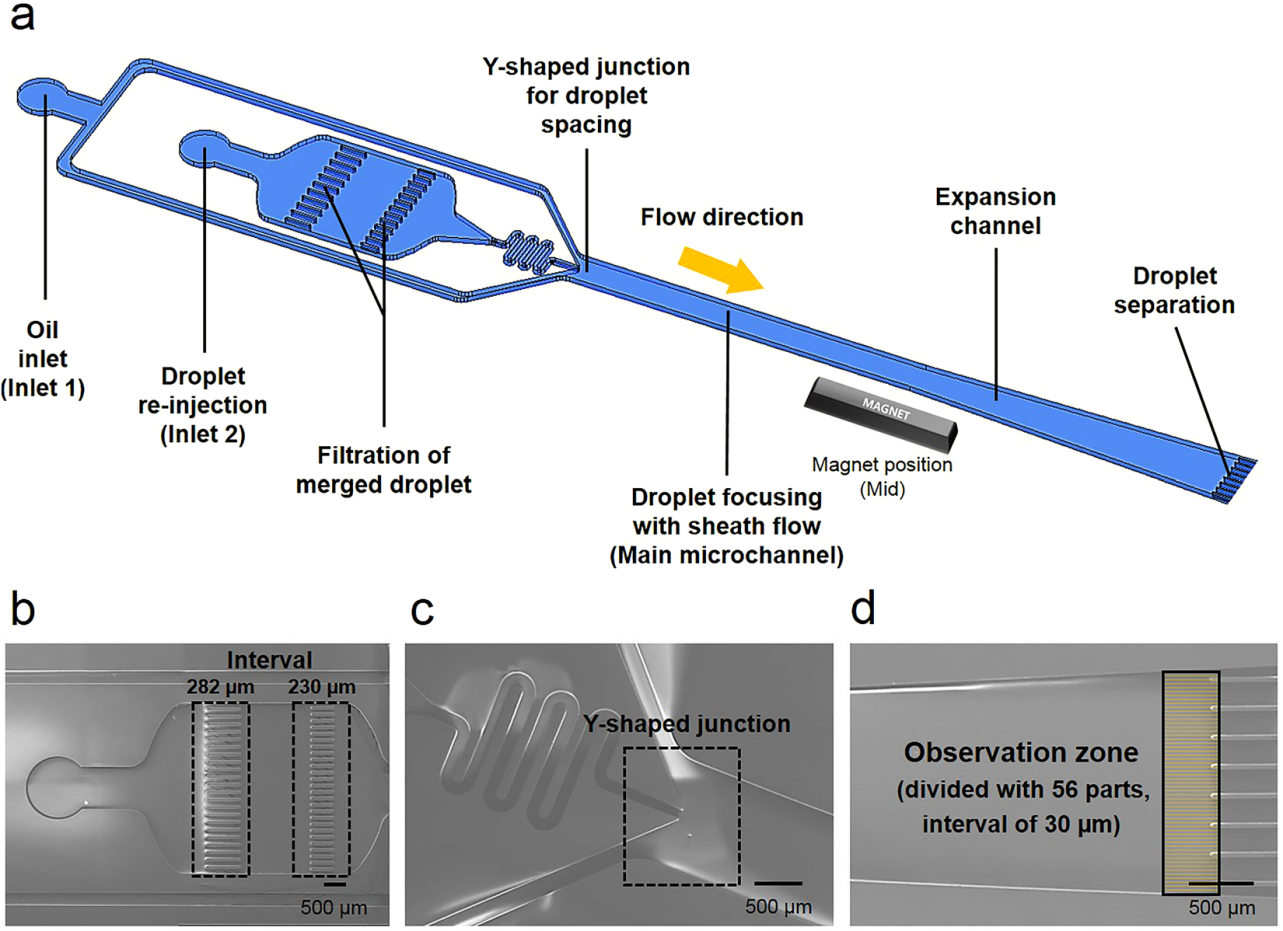
Design of the microfluidic device to magnetically separate the microdroplets and scanning electron microscope (SEM) images of the three parts of the microfluidics. (**a**) There are three *x*-axis positions of the magnet determined by the distance between the magnet and the Y-shaped junction, such as the front, mid, and end. The magnetic force can act on the magnetic droplets in the perpendicular direction due to the magnetic field during droplet flow in the microchannel. In addition, there is a sudden expansion in the channel to increase the separation efficiency. (**b**) Magnetic droplets from inlet 2 are injected into microchamber containing micropillar arrays to filter the merged droplets. (**c**) Microdroplets with a diameter of ~160 µm are focused on the central flow and spaced by oil phase from inlet 1. (**d**) At the end of the microchannel, there are seven outlets to recover the separated droplets.

Microdroplets with 160-µm diameters were generated to contain three different concentrations of cell suspension (Supplementary Fig. S1). For separation, the generated microdroplets were reinjected into inlet 2 in the microfluidic device through polyethylene (PE) tubing using a pressure pump (Fig. 2a). The reinjected microdroplets can merge with each other to form large microdroplets (>160 µm) due to pressurization. These merged droplets can clog the microchannel and obstruct the flow of the oil and aqueous phase. For this reason, a micropillar structure was integrated into the microfluidic device to filter the merged droplets (Supplementary Fig. S2). The intervals between the micropillars were set to 282 µm and 230 µm for two-step sequential filtration (Fig. 2b). The fraction of merged droplets decreased from 5% to 1.67% by introducing the micropillar structure into the microchamber (n = 120). The filtered microdroplets from the microchamber were focused toward the center of the streamline at the Y-shaped junction (Fig. 2c). The FC-40 oil from inlet 1 is divided and injected into two paths of microchannels to produce the sheath flow. Therefore, a laminar flow consisting of the sheath and core microdroplet fluids is generated in the 1,055 µm width of the main microchannel (Fig. 2c). In addition, the microdroplets can be spaced by an oil phase to avoid the interference of the neighboring droplets, which can disturb the deflection path of the microdroplets. To apply a magnetic field on the magnetic microdroplets in the perpendicular direction of the microchannel, a permanent magnet is placed at the side of a microfluidic chip. It is possible to regulate the magnetic flux density on the microdroplet by adjusting the *x*- and *y*-a*x*is positions of the magnet determined by the distance from the Y-shaped junction and the main microchannel, respectively (Fig. 2a). The microdroplets can be deflected by the magnetic force in the main microchannel. However, differences in the lateral displacement are too small to separate the microdroplets into different outlet sub-microchannels. To overcome this limitation, the sudden expansion of the microchannel was introduced to increase the *y*-axis distance between the microdroplets. The expansion angle and the length of the channel were set at 2 degrees and 5,000 µm, respectively, which can maintain the laminar flow and stable flow of the microdroplets. After the microdroplets go through the expansion channel, the *y*-axis distance among the microdroplets can increase by 59.2%. At the end of the expansion channel, there is an observation region that is divided into 56 virtual parts at intervals of 30 µm to investigate the effects of various parameters on the amount of lateral displacement of the droplets and 7 outlets to recover the droplets (Fig. 2d).

### Investigation of the magnetic flux density and effect of the iron oxide nanoparticle concentration on the deflection degree of the droplets

**Figure 3:**
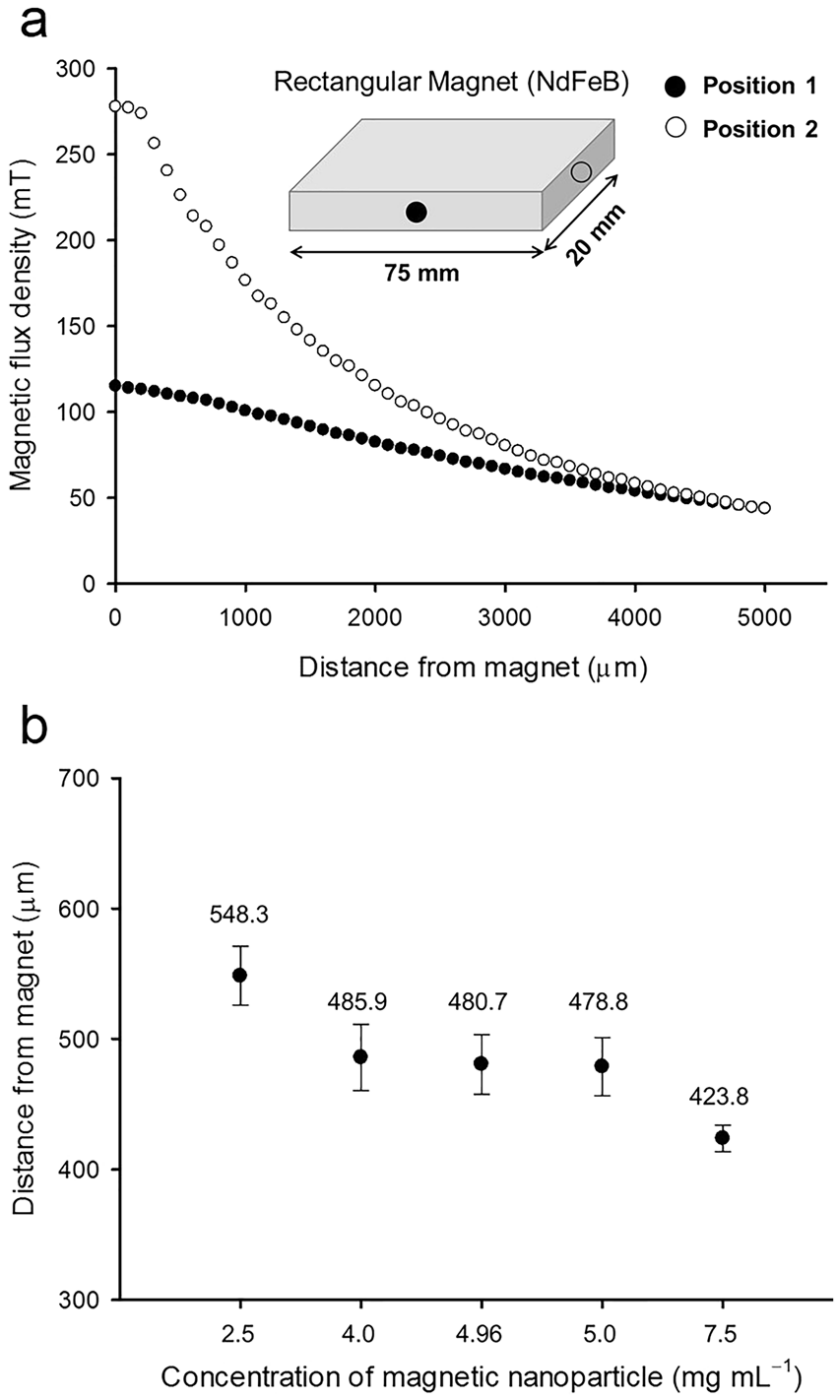
Effects of three parameters (distance from the magnet, side position of the magnet, and magnetic nanoparticle concentration in microdroplets) on the magnetic force. (**a**) Measured magnetic flux density (mT) at a given distance from two different sides of the magnet. The rectangular magnet can have two different sides (position 1 and 2) showing different distribution of the magnetic field. The magnetic flux density at position 2 is higher than that at position 1 with distance range between 0 and 5,000 µm. (**b**) Microdroplets containing different concentrations of magnetic nanoparticles (2.5, 4.0, 4.96, 5.0 and 7.5 mg mL^−1^) are deflected toward different positions which can be determined by the distance from magnet. Data and error bars are mean ± SD (standard deviation) of three replicates.

A permanent NdFeB magnet was used to apply a magnetic field to the magnetic nanoparticles in the microdroplets. The magnetic field generated by the magnet contributes to the magnetization of the magnetic nanoparticles. From equation (1), the magnetic force can be determined by the magnetic flux density (*B*) and magnetic flux density gradient (∇*B*). We investigated the magnetic flux density (*B*) at a given distance from the magnet surface, and the magnetic flux distributions from two different sides of the magnet are expected to be different due to the rectangular shape of the magnet. As the distance from the magnet increases, the magnetic flux density (*B*) and its gradient (∇*B*) decrease but the degrees of the slopes of the magnetic flux density from two different sides of the magnet were different (Fig. 3a). The magnetic flux density from position 2 of the magnet was higher than that at position 1. Thus, we concluded to use the magnet at position 2 to improve the efficiency of the separation of the microdroplets with different cell densities (Fig. 3a).

When microdroplets with a diameter of 160 µm were generated from an aqueous phase containing magnetic nanoparticles (5.0 mg mL^−1^), the volume of microdroplet is about 2 nL. When generating 160 µm of high-density droplet which contains 2 µm sized cells with concentration of 2.0 × 10^9^ cells mL^−1^, the total cell volume in droplet is about 0.016 nL (0.8% of total droplet volume). Accordingly, the final concentrations of the magnetic nanoparticles in the empty and high-density droplet are 5.0 and 4.96 mg mL^−1^, respectively, because the volume of the high-concentration microalgal cells accounts for 0.8% of the total microdroplet volume. We investigated the effects of the concentrations of the magnetic nanoparticles in the microdroplet (7.5, 5.0, 4.96, 4, and 2.5 mg mL^−1^) on the deflection angles to confirm whether the decrease in the number of magnetic nanoparticles for a given cell volume in the microdroplet was critical in deflecting the microdroplets. Although the microdroplets moved more toward the magnet as the concentration of magnetic nanoparticles increased from 2.5 to 7.5 mg mL^−1^ due to the increase in the magnetic force, the differences in the distance from the magnet among microdroplets containing 4, 4.96, and 5 mg mL^−1^ were not significant (Fig. 3b). Consequently, the decrease in the magnetic nanoparticle concentration caused by the volume occupied by the microalgal cells in the microdroplet was not critical in changing the deflection degree of the magnetic microdroplets. In conclusion, the lateral displacement of the droplet is determined by the difference in density, not the difference in the number of magnetic nanoparticles and the magnetic force.

### Effect of four different parameters on magnetophoretic separation efficiency

**Figure 4:**
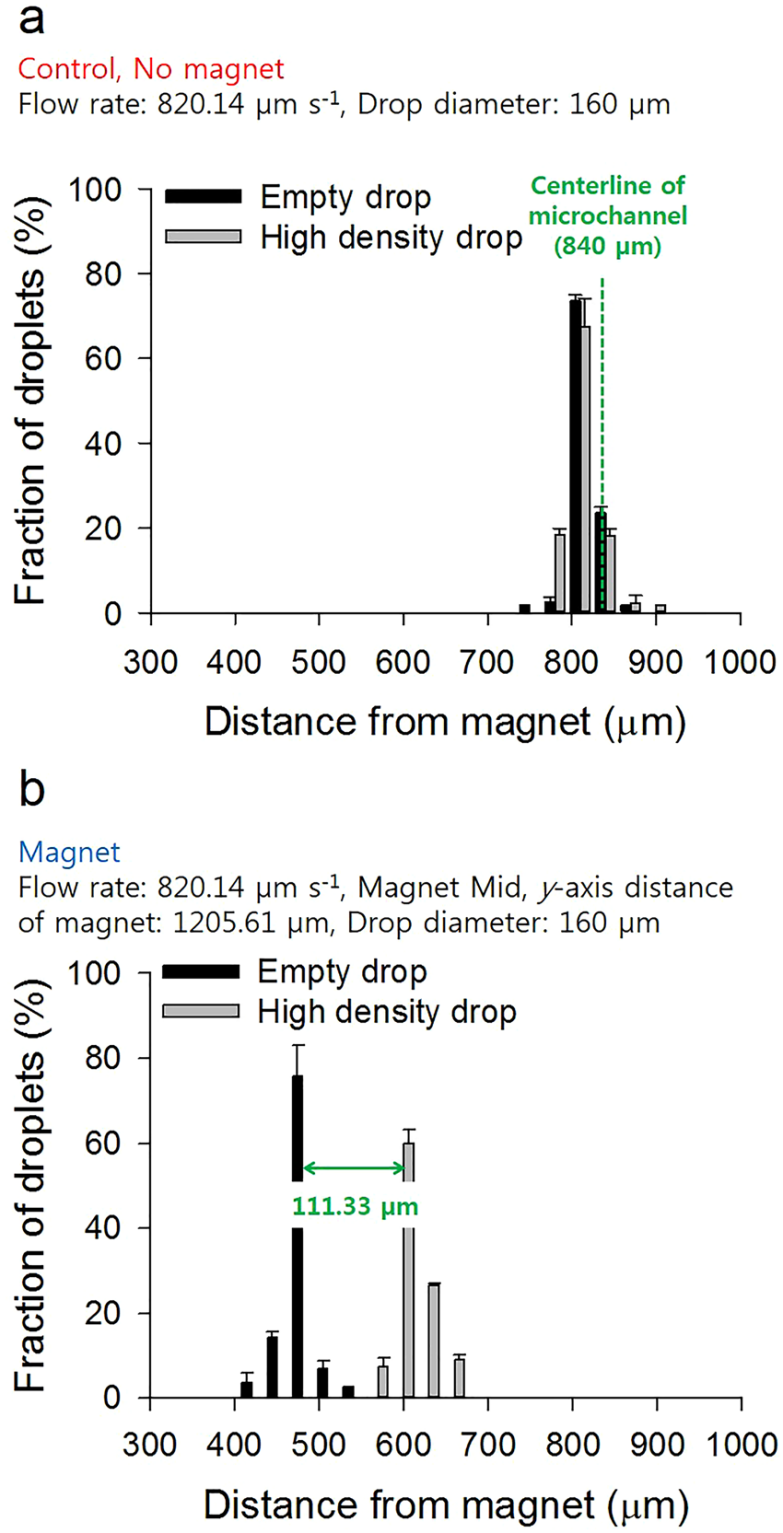
Distribution of deflected microdroplets without or with the magnet. (**a**) There is no magnet near the microfluidic channel. 73.5% and 67.4% of the empty and high-density droplets (diameter of 160 µm), respectively, pass through the point near the centerline of microchannel (840 µm away from the magnet) with the flow rate of 820.14 µm s^−1^. There is little gap between the empty and high-density droplets. (CONTROL) (**b**) The magnet is located 1205.61 µm away from and the mid position of the main microchannel. 75.8% of the empty and 60.0% of the high-density droplets (diameter of 160 µm) pass through the point where the distance from the magnet is 480 µm and 600 µm, respectively. Fraction data and error bars are mean ± SD (standard deviation) of three replicates.

We investigated the effects of four different parameters, such as the flow rate of the main stream, the magnet position (*x*- and *y*-axis) and the droplet diameter, on the separation of two kinds of microdroplets with different cell concentrations (0, 2.0 × 10^9^ cells mL^−1^) using a magnetic field. 73.5% of empty and 67.4% of high-density microdroplets can pass through a point 30 µm away from the centerline of the microchannel without a magnet (Fig. 4a). As the flow rate of microdroplets decreased, a high fraction of the microdroplets moved away from the centerline due to the increased exposure time to the magnetic field (Supplementary Fig. S3a). When the flow rate of microdroplets reached 820.14 µm s^−1^, the maximum gap between the empty and high-density droplets was created (111.33 µm) (Fig. 4b, Supplementary Table S1). To determine the optimal position of the magnet, the lateral displacement of the droplet was investigated according to *x*-axis distance of the magnet from the Y-shaped junction (from 0 to 21.0 mm). When the magnet was located at the front position, the distribution of the empty and high-density microdroplets overlapped due to an inefficient separation of the droplets (Supplementary Fig. S3b). As the *x*-axis distance between the Y-shaped junction and the magnet gradually increased, the empty droplets were attracted more to the magnet compared to the droplets with high cell densities (Supplementary Fig. S3b). Therefore, the optimal position of the magnet as a starting point of the expansion channel (mid position) and the maximum separation efficiency of the droplets were determined (Fig. 4b). As shown in Fig. 3a, the gradient of the magnetic flux density decreased as the *y*-axis distance increased from the magnet, allowing us to control the magnetic force by adjusting the *y*-axis distance. When the magnet is located near the microchannel at a *y*-axis distance of 527.81 µm, the fractions of the empty and high-density droplets that deflected toward the point 720 µm away from the centerline are 97.2% and 97.1%, respectively (Supplementary Fig. S3c). The gradual increase in the *y*-axis distance between the microchannel and the magnet caused a reduction in the deflection angle of the microdroplets due to the decrease in the magnetic force. Consequently, when the *y*-axis distance between the magnet and the centerline of the microchannel was 3781.25 µm, 48.6% and 45.8% of the empty and high-density microdroplets moved into the position, 60 µm and 90 µm away from the center, respectively, showing similar deflection pattern of droplets without the magnet (Supplementary Fig. S3c). From equation (3), the net force on the magnetic microdroplet was expressed by the difference between the magnetic and the drag force. The deflection angle of the microdroplet was affected by the total number of magnetic nanoparticles (*N*) and the radius of microdroplets (*r*). We investigated the movement of two different microdroplets of 80 µm and 160 µm in diameter with the same concentration of magnetic nanoparticles (5 mg mL^−1^). The larger droplet (160 µm) contains 8 times more magnetic nanoparticles than the smaller one (80 µm) due to the relationship between the radius (*r*) and volume (*V*) (*r* ^3^ ∝ *V*). Thus, a high proportion of the large empty droplet (75.8%) was deflected toward the position (360 µm away from the centerline) compared to the small empty droplet (55.1%) (Supplementary Fig. S3d). On the other hand, in terms of the number of cells encapsulated by the droplet, the large high-density droplet had a mass much larger than that of the small high-density droplet because all droplets have the same concentration of cells with 2.0 × 10^9^ cells mL^−1^. Therefore, 60.0% of the large and 45.5% of the small high-density droplets passed through the points, 240 and 300 µm away from the centerline, respectively (Supplementary Fig. S3d). In other words, the deflection angle of the large droplet containing cells was relatively smaller than that of the small droplet. In conclusion, we could increase the distance between the empty and high-density droplets by using droplets with a diameter of 160 µm instead of 80 µm. In conclusion, optimum conditions for efficient magnetophoretic separation were determined as follows: flow rate (µm s^−1^), 820.14; position of the magnet, mid; *y*-axis distance of the magnet (µm), 1205.61; diameter of droplet (µm), 160, respectively.

### Magnetic separation of empty, low-, and high-density microdroplets with stepwise expansion of the microchannel

**Figure 5:**
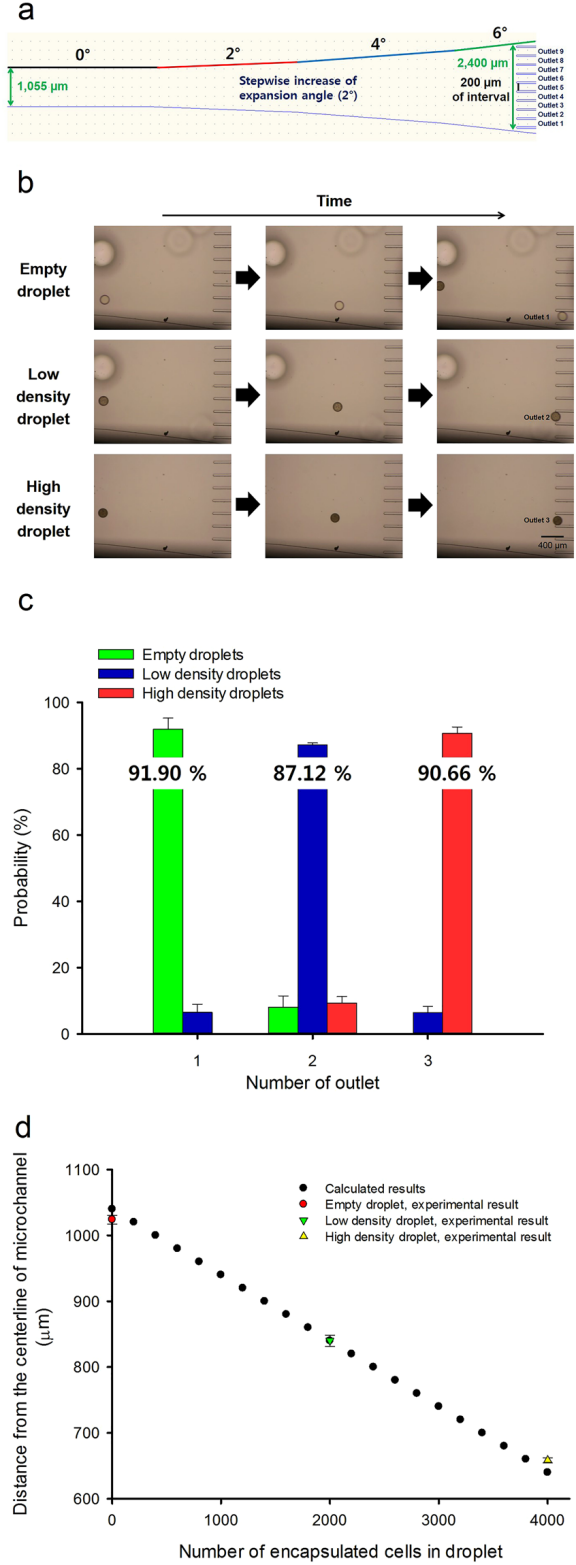
Separation of three different magnetic microdroplets (empty, low-density, high-density). (**a**) An expansion microchannel with stepwise increase of the expansion angle (2, 4, and 6 degrees) is introduced to increase the gap between the droplets at a separation region having 9 outlets. Width and length of the expansion microchannel are 2,400 µm at the observation region and 10,000 µm, respectively. (**b**) To investigate the migration of the droplets, the images were taken at 1.34-s interval. Magnetic microdroplets containing different densities of cells showed different migration routes on the magnetic field. (**c**) Distribution of separated droplets in accordance with the outlet position (n = 60). Probability data and error bars are mean ± SD (standard deviation) of three replicates. (**d**) Comparison of calculated and experimental results of distance from the centerline of microchannel according to the number of encapsulated cells in droplet. Experimental data and error bars are mean ± SD (standard deviation) of three replicates.

To separate the magnetic microdroplets with three different cell densities, it was necessary to increase the gap between the droplets. As shown in Fig. 4b, 111.33 µm of the gap between the droplets was sufficient to separate the empty and high-density droplets, but was insufficient to separate the empty, low-, and high-density droplets. Therefore, the existing expansion microchannel was re–designed to have a stepwise increase in the expansion angle, such as 2, 4, and 6 degrees and 10,000 µm of the length. In addition, the separation region has 9 outlets with a 200 µm interval where only one droplet with a diameter of 160 µm could pass (Fig. 5a). Therefore, the width of the microchannel increased from 1,055 µm at the starting point of the expanded microchannel to 2,400 µm at the observation region. In stable laminar flow, the gap between the droplets can increase by 127.5% (Fig. 5a), which is higher than the increase in the gap in the existing expansion of the microchannel (59.2%) (Fig. 2d). Consequently, the magnetic microdroplets with a diameter of 160 µm containing a different number of cells could be separated in stepwise expanded microchannel with optimum separation conditions described in Fig. 4b. It is worth noting that the separation of more than two kinds of microdroplets based on the density-dependent magnetophoretic sorting had been made possible for the first time utilizing stepwise widening of the microchannel. To track the migration route of the droplets, images were taken at 1.34-s interval when the droplet passed through the observation zone (Fig. 5b). We observed that 91.90%, 87.12%, and 90.66% of the empty, low-, and high-density droplets passed through outlets 1, 2, and 3, respectively (n = 60) (Fig. 5c, Table 1), which indicate that it was possible to separate three kinds of microdroplets with different cell densities in the microfluidic system using a permanent magnet (Supplementary Video S1). To investigate the sensitivity of this technology, we calculated the simulation data for lateral displacement of the magnetic droplets based on the equation (6) described in Results section of Theoretical analysis part. For the calculation, we considered that the mass of a single living microalgal cell (*Chlorella sorokiniana* (formerly *Chlorella vulgaris*)) is about 10 pg^34^. In addition, we could get experimental results from investigation of distribution of separated droplets in accordance with the outlet position based on the statistical analysis (Table 1). The experimental results were matched well with the theoretical calculation and demonstrated that as the number of encapsulated cells in droplet decreased, the droplets moved away from the center of the microchannel (Fig. 5d).

**Table 1:** Separation fraction of three magnetic microdroplets with different cell densities in different outlets. Probability data and error bars are mean ± SD (standard deviation) of three replicates.

	Empty droplet	Low-density droplet	High-density droplet
Outlet 1	91.90 ± 3.37%	6.50 ± 2.47%	—
Outlet 2	8.10 ± 3.37%	87.12 ± 0.66%	9.34 ± 1.90%
Outlet 3	—	6.38 ± 1.90%	90.66 ± 1.90%

## Discussion

In this work, we developed a microfluidic system that uses a magnetic force to separate the three kinds of magnetic microdroplets containing a different number of microalgal cells. Although a magnetic force has been used to manipulate, generate, and split microdroplets in the microfluidic device, there have been no reports on density-based separation of the droplets using a magnetic force. This density-based magnetophoretic sorting system has been made possible for the first time using a featured design of microchannel in microfluidic device, which enables distinguishing micrometer-scale difference in lateral displacement. Therefore, we can isolate the high-density microdroplet photobioreactors containing fast growing microalgal strains which are essential for the commercial use of microalgae having great potential to convert CO_2_ into biomass and high-value products. By encapsulating dextran-coated superparamagnetic magnetite (Fe_3_O_4_) nanoparticles, droplets containing microalgal cells can be attracted toward the permanent NdFeB magnet, and the same net force exerted on the magnetic droplet results in different acceleration and lateral displacements in the *y*-axis due to differences in the mass of the droplet. This approach is distinguished from previous magnetophoretic sorting system^31, 32^ which depends on difference in the amount of the magnetic force caused by the different number of encapsulated magnetic nanoparticles. To maximize the gap between the droplets, we investigated the lateral displacement of the deflected droplets by regulating four different parameters, including the flow rate of the main stream, the magnet position (*x*- and *y*-axis) and the droplet diameter. Furthermore, a microchannel was designed to have a stepwise increase in the expansion angle to efficiently separate three kinds of magnetic microdroplets with different cell densities. Consequently, we could recover 91.90% of empty, 87.12% of low-density, and 90.66% of high-density droplets at outlets 1, 2, and 3, respectively. Our results indicate that the cell density-based separation of the microdroplets using the magnetic force can provide a promising platform to isolate microalgal species with a high growth performance. As described in Supplementary Fig. S4a, when we used the microdroplet as a photobioreactor for cultivation of microalgae, about 2,000 of wild-type cells of *C. sorokiniana* could be grown in the microdroplets under photoautotrophic conditions. Given these cell growth results, if this magnetophoretic sorting system is applied to a mutant library, we will be able to obtain the strains having a growth rate more than twice that of a wild-type strain. These superior strains will be expected to be a great help to overcome low biomass productivity, which is a hurdle to commercialization of microalgae culture process. However, there was a limitation on the separation of droplets with a smaller mass difference than a mass of 2,000-microalgal cells. To overcome this limitation, it is necessary to increase the magnetic force affected by the number (*N*), volume (*V*_*p*_) of the magnetic nanoparticles, magnetic flux density (*B*) and its gradient (*∇B*). There was a previous report on the use of a nickel microstructure near the magnet or an electromagnet to increase the magnetic flux density gradient^32^. Although there is limitation to applying this system to large sized microalgal strains due to the changes in the magnetic force, our platform can be applied to other useful microalgal strains which have much smaller cell size than the droplet and a great potential to be utilized as biofuel and high-value products.

## Methods

### Fabrication of microfluidic devices

The microfluidic devices used to generate and separate the microdroplets were designed using the AutoCAD software (Autodesk, USA) and were printed on photomask films (Han & All Technology, Korea). Two master molds were fabricated on silicon wafers coated with a negative photoresist, SU-8 50 (Microchem, USA) using standard photolithography^35^. To prepare the PDMS prepolymer, Sylgard 184 and a curing agent were mixed at a ratio of 10:1 (w/w). This prepolymer was poured onto the master molds and was degassed using a desiccator and a vacuum pump. After curing in an oven at 80 °C for 12 hours, PDMS slabs were peeled off from the master molds using a sharp scalpel and punched with a biopsy punch with a diameter of 1 mm to make the inlet and outlet holes. PDMS slabs and glass slides were covalently bonded using an oxygen plasma treatment. The fabricated PDMS microfluidic devices were placed into an oven at 120 °C overnight to recover the hydrophobicity.

### Generation of the microdroplets

Fluorocarbon oil FC-40 (Sigma-Aldrich) with 5% (w/w) perfluorinated polyethers (PFPE) and a polyethyleneglycol (PEG) block copolymer surfactant (Ran Biotechnologies, Beverly, MA) were used as an oil phase. This biocompatible surfactant can provide excellent drop stability against coalescence^36^. Dextran-coated superparamagnetic magnetite (Fe_3_O_4_) nanoparticles (Micromod Partikeltechnologie GmbH, Rostock, Germany) and 2–5 µm sized microalgal cells of *Chlorella sorokiniana* (formerly *Chlorella vulgaris*) UTEX 2714 were suspended in Tris-phosphate (TP) medium as an aqueous phase. To make magnetic microdroplets with different cell densities, a cell suspension was prepared at 0, 1.0 × 10^9^, and 2.0 × 10^9^ cells mL^−1^ for empty, low-, and high-density droplets, respectively, containing the same number of magnetic nanoparticles. The size distributions of generated droplets were investigated using ImageJ software. Diameters of empty, low-density, and high-density droplets were 158.42 ± 1.03 µm (n = 118), 158.94 ± 1.27 µm (n = 109), and 159.04 ± 0.76 µm (n = 136), respectively (Supplementary Fig. S1). The oil and aqueous phases were loaded into 1.5 mL reservoirs connected with a pressure-driven pump (Fluigent, France) and a microfluidic device through the polyethylene (PE) tube. The microfluidic device contains a flow-focusing microchannel of 60 µm × 100 µm (width × height) to generate the microdroplets. Microdroplets with a diameter of 80 µm and 160 µm were formed setting the injection pressure to 80 mbar/160 mbar and 150 mbar/160 mbar (aqueous phase/oil phase), respectively. After generation, the microdroplets passed through the long serpentine microchannel to increase the stability of the microdroplets due to the well dispersion of the surfactant caused by improved the mixing performance (Supplementary Fig. S1)^37^. We collected each types of droplets getting out from the outlet of microfluidic device. All droplets were transferred from the outlet of droplet generation device to 1.5 mL eppendorf tube to make a droplet mixture.

### Preparation and photoautotrophic cultivation of microalgal cells in microdroplet photobioreactor

*Chlorella sorokiniana* (formerly *Chlorella vulgaris*) UTEX 2714 was obtained from the Culture Collection of Algae at the University of Texas. The microalgal cell suspension was diluted to 2.6 × 10^6^ cells mL^−1^ with TAP-C medium and mixed with 5 mg mL^−1^ of dextran-coated superparamagnetic magnetite (Fe_3_O_4_) nanoparticles solution. The 160 µm sized droplets were generated using droplet generation device (Supplementary Fig. S1) and reinjected into the droplet incubation device (Supplementary Fig. S4b). For the photoautotrophic cultivation of microalgal cells in microdroplet, the droplet incubation device was stored at 23 °C with 5% CO_2_ in a CO_2_ incubator under continuous light of 150 µmol photons m^−2^ s^−1^. In addition, the humidity of CO_2_ incubator was maintained at 90% to prevent droplet shrinkage^25^. The images of cells in microdroplet was taken by DSLR camera. We could count the number of cells in microdroplet using the naked eye and ImageJ software.

### Investigation of the magnetic flux density at a given distance from the magnet

The magnetic field was produced using a permanent NdFeB magnet (LG magnet, Korea) with a size of 75 mm × 20 mm × 6 mm (length × width × height). The magnetic flux density was measured at two positions of the magnet (position 1 and 2, Fig. 3a) using a gaussmeter (Model MG-3003SD, Lutron, Taiwan) programmed with a measuring time of 1 second. The magnet was gradually moved away from the measuring probe of the gaussmeter at a rate of 100 µm s^−1^. Thus, we could investigate the magnetic flux density according to the distance from the magnet at different positions.

### Investigation of the deflection degree of microdroplets with different magnetic nanoparticle concentrations and microalgal cell densities

The microfluidic device that was used to separate the magnetic microdroplets was comprised of four chambers: filtration, focusing, expansion, and separation. The microdroplets generated with different cell densities were reinjected into the microchamber containing two types of micropillar arrays with different intervals (230 µm and 282 µm, Fig. 2a and b), which could filter the merged droplets. Reinjected microdroplets were spaced and focused by an oil phase at a Y-shaped junction (Fig. 2c). The sheath flow of the oil phase permits the microdroplets to move along the center streamline in which a uniform magnetic field is applied in the perpendicular direction to the streamline. To investigate the effect of the magnetic nanoparticle concentration on the deflection degree of the microdroplets, an aqueous phase was prepared with different magnetic nanoparticle concentrations (7.5, 5.0, 4.96, 4, and 2.5 mg mL^−1^). To change the flow rate of the magnetic microdroplets, the injection pressures of the microdroplets and the oil phase were regulated using a pressure pump. Thus, a flow rate of 820.14 µm s^−1^ was determined when the microdroplets and oil phase were injected at a pressure of 20 and 40 mbar, respectively. There were three *x*-axis positions, including front, mid and end (distance of 0 mm, 10.5 mm, and 21 mm from the Y-shaped junction) and five *y*-axis positions determined by the distance between the magnet and the main microchannel (from 527.81 µm to 3781.25 µm) to control the magnetic field, which could affect the deflection angle of the magnetic microdroplets. To increase the gap between the droplets, a sudden expansion in the microchannel was constructed with an expansion angle of 2 degrees. At the end of the microchannel, there was an observation zone in which the fraction of separated microdroplets was analyzed. An observation channel was divided into 56 virtual parts (interval of 30 µm) to investigate the effects of the different parameters on the amount of lateral displacement of droplets (Fig. 2d). In addition, the sudden expansion in the microchannel was re–constructed to have a stepwise increase in the expansion angle (2, 4, and 6 degrees) with 9 outlets, numbered from 1 (near the magnet) to 9, to separate the microdroplets with three different densities (Fig. 5a).

### Image acquisition and data analysis

The microchannel structures fabricated in the microfluidics were verified by taking scanning electron microscope (SEM) images using a Zeiss Supra 55 VP field-emission scanning electron microscope (FESEM) (Carl Zeiss, Germany). Images of the generated and separated microdroplets were taken on an inverted microscope (Olympus KX41) using a DSLR camera (Canon EOS 700D). To investigate the deflection pattern of the microdroplets with different densities into separated outlets in the observation zone, images were captured at 1.34-s intervals. In addition, the movement of the microdroplets was monitored and recorded using a digital video camera. A fraction of the deflected microdroplets was determined by counting the number of microdroplets using the naked eye. It was possible to distinguish empty, low-, and high-density microdroplets based on their color intensity due to the different number of cells encapsulated into the microdroplets (Supplementary Fig. S1).

## Electronic supplementary material

Supplementary Information

Supplementary Video S1
