# Supplementary material for: Magnetophoretic sorting of microdroplets with different microalgal cell densities for rapid isolation of fast growing strains
Source: Sci Rep. 2017 Sep 4;7:10390. doi: 10.1038/s41598-017-10764-6 (PMC5583291; doi:10.1038/s41598-017-10764-6)
Supplement: Supplementary file 1 — Supplementary Information [file 41598_2017_10764_MOESM1_ESM.pdf]

Electronic Supplementary Information for

Magnetophoretic sorting of microdroplets with different microalgal cell densities for rapid isolation of fast growing strains

Young Joon Sung[1, †], Jaoon Young Hwan Kim[2, †], Hong Il Choi[1], Ho Seok Kwak[3], and Sang Jun Sim[1, *]

[1]Department of Chemical and Biological Engineering, Korea University, Seoul 136-713, Republic of Korea.

[2]Convergence Research Division, National Marine Biodiversity Institute of Korea, Jangsan-ro 101beon-gil 75, Janghang-eup, Seocheon-gun, Chungcheongnam-do 33662, Republic of Korea.

[3]Department of Food Engineering, Dongyang Mirae University, 445, Gyeongin-ro, Guro-gu, Seoul, 08221, Republic of Korea.

*Corresponding author: Professor Sang Jun Sim (email: simsj@korea.ac.kr)

[†]These authors contributed equally to this work.

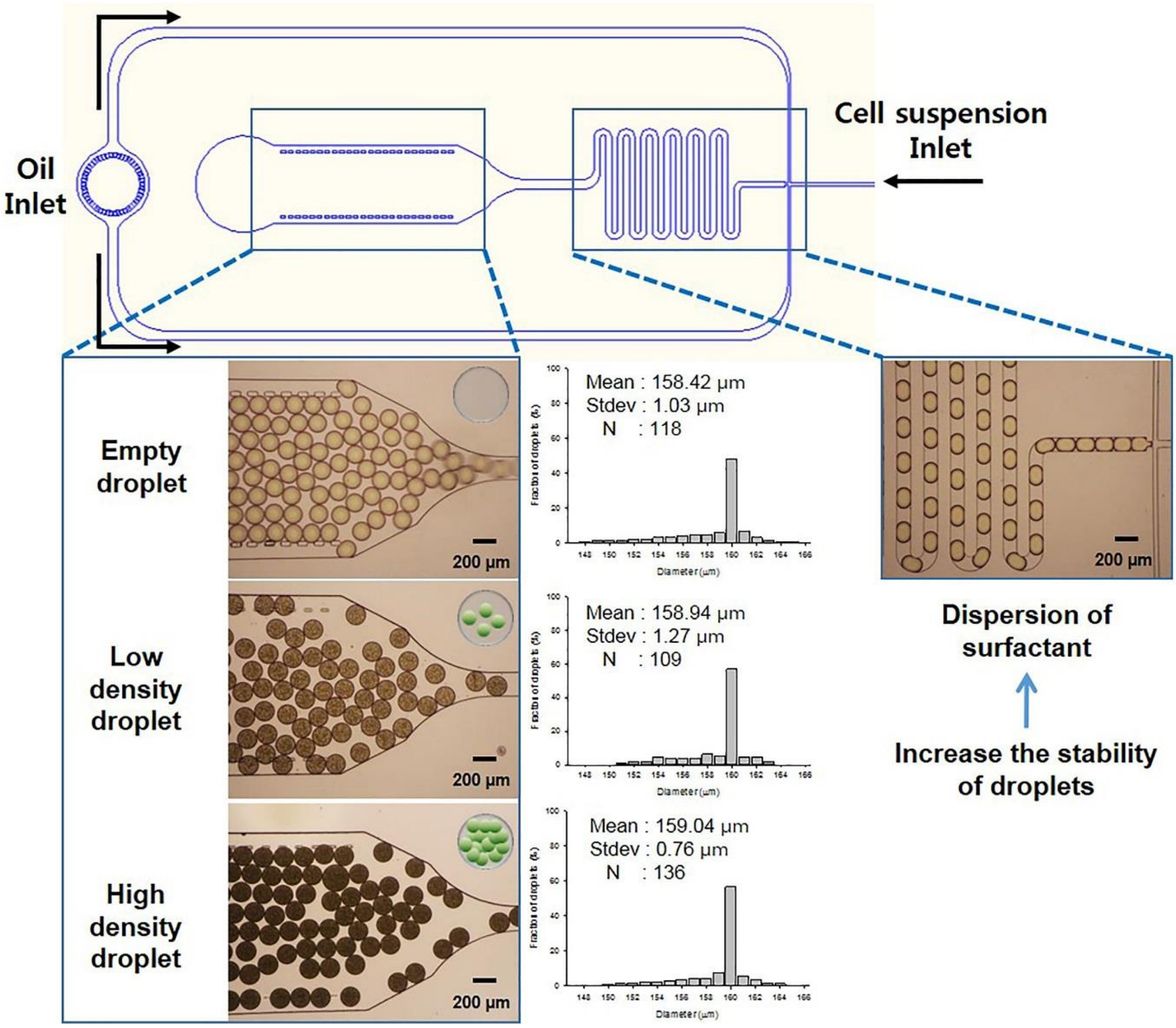

Supplementary Figure S1. Microfluidic device design used to generate the magnetic microdroplets. Two immiscible phases (oil and cell suspension (0, 1.0 × 10^9, 2.0 × 10^9 cells mL^{-1}) containing magnetic nanoparticles (5 mg mL^{-1})) are co-injected into the flow-focusing channel to form the droplets. Diameters of empty, low-density, and high-density droplets were 158.42 ± 1.03 μm (n = 118), 158.94 ± 1.27 μm (n = 109), and 159.04 ± 0.76 μm (n = 136), respectively. In the long serpentine channels, surfactant can be well-dispersed on the surface of the droplets to increase the stability of the droplets.

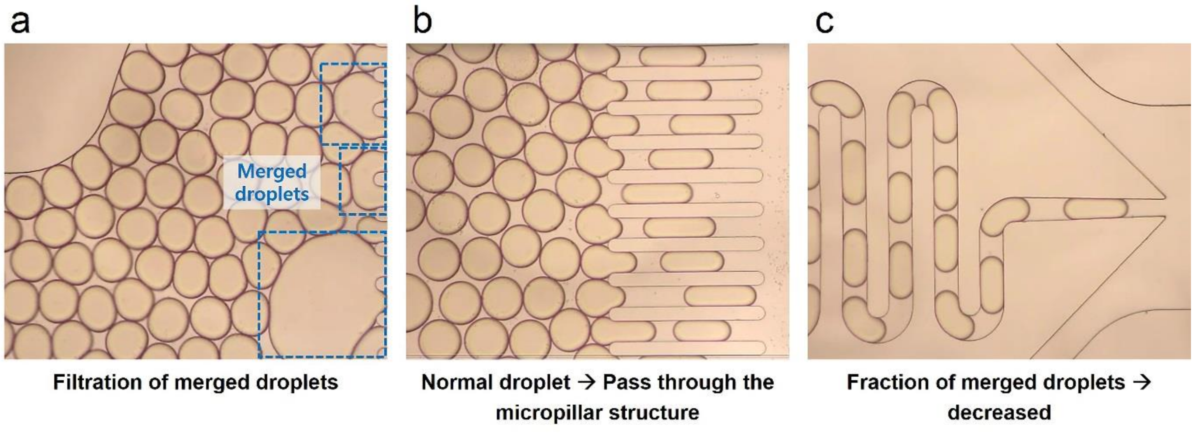

Supplementary Figure S2. Fraction of merged droplets when the generated droplets are reinjected into the microchamber. (a) The fraction of merged droplets is about 5% (n = 120) before reinjected droplets pass through a micropillar structure. These merged droplets which can clog the microchannel are filtered by micropillar structure in microchamber. (b) Normal droplets (~160 μm of diameter) can pass through the micropillar structure. (c) After the reinjected droplets passed through the micropillar structure in microchamber, the fraction of merged droplets decreased to about 1.67% (n = 120). We can conclude that introducing the micropillar structure into the microchamber is effective in removing merged droplets.

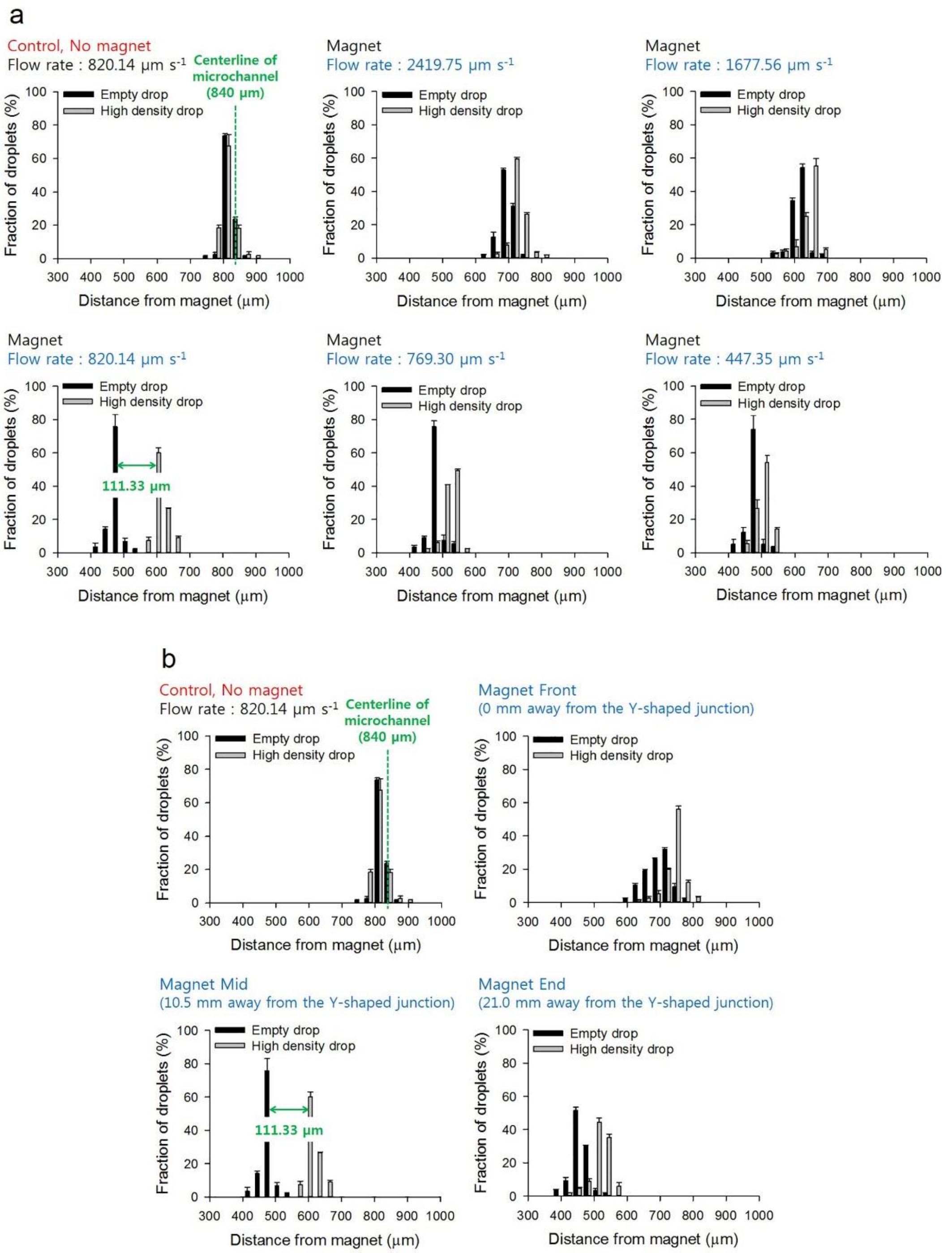
a
Control, No magnet
Flow rate : 820.14 μm s-1
Centerline of microchannel (840 μm)
Magnet
Flow rate : 2419.75 μm s-1
Magnet
Flow rate : 1677.56 μm s-1
Magnet
Flow rate : 820.14 μm s-1
111.33 μm
Magnet
Flow rate : 769.30 μm s-1
Magnet
Flow rate : 447.35 μm s-1
Empty drop
High density drop
Fraction of droplets (%)
Distance from magnet (μm)
b
Control, No magnet
Flow rate : 820.14 μm s-1
Centerline of microchannel (840 μm)
Magnet Front
(0 mm away from the Y-shaped junction)
Magnet Mid
(10.5 mm away from the Y-shaped junction)
111.33 μm
Magnet End
(21.0 mm away from the Y-shaped junction)
Empty drop
High density drop
Fraction of droplets (%)
Distance from magnet (μm)

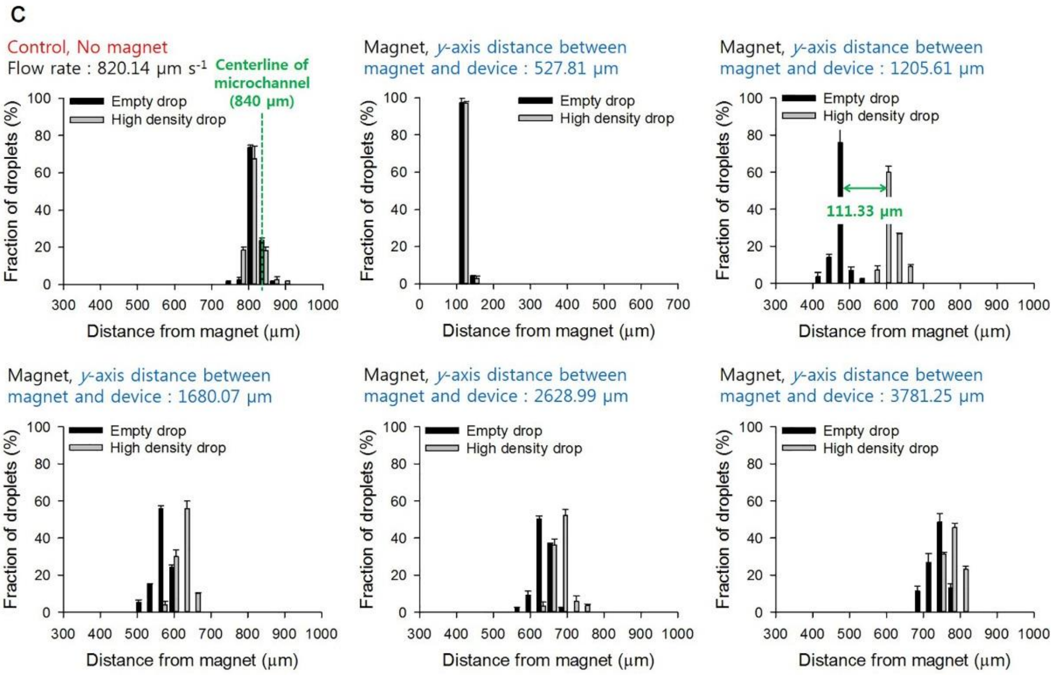

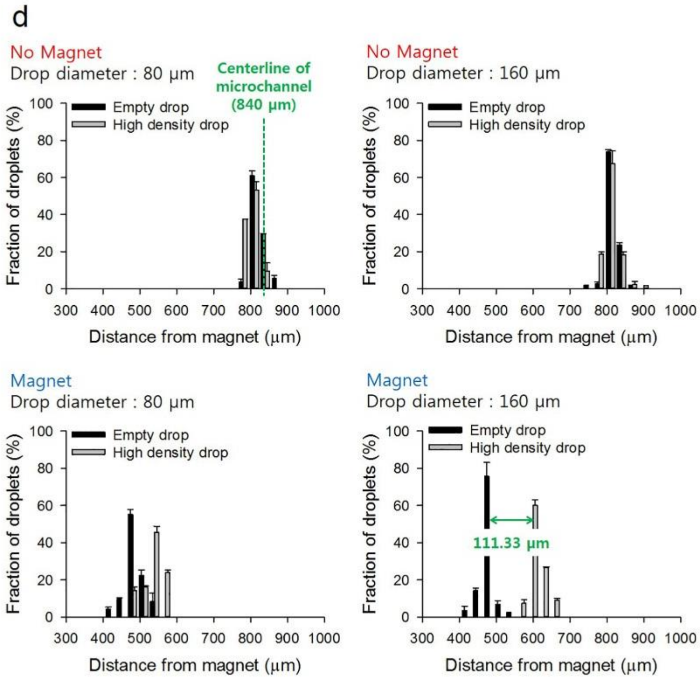

Supplementary Figure S3. Distribution of deflected microdroplets according to various parameters, such as the flow rate of droplets, the magnet position (*x*- and *y*-axis) and droplet diameter. (a) Exposure time to magnetic field decreases as flow rate of magnetic droplet increases. A lower flow rate induces more attraction of the magnetic droplets toward the magnet. Among different flow rate conditions, flow rate of 820.14 μm s^{-1} causes maximum separation

efficiency of the droplets. (b) There are three kinds of *x*-axis magnet positions: front, mid, and end position. Magnetic droplets are inefficiently separated by locating the magnet at the front or end position. To maximize the gap between the droplets, it is necessary to locate the magnet at the mid position. (c) The distance between the magnet and microfluidic device can affect the amount of the magnetic force. As the distance increases or the magnetic force decreases, the deflection angle of the droplets decreases. Empty, high-density droplets can be separated efficiently with 1205.61 μm of distance between the magnet and microchannel. (d) When the diameter of the droplet is about 80 μm, it is difficult to separate the empty and high-density droplets. In conclusion, an optimal diameter of the droplet for separation is of about 160 μm. We performed each experiment with the optimum separation conditions (820.14 μm s^{-1} of flow rate, mid position of the magnet, 1205.61 μm of *y*-axis distance of the magnet, 160 μm of droplet diameter) except for one parameter which is required to be investigated. Deflection patterns of droplets ($n \geq 40$) were investigated to determine the fraction of droplets (%). Fraction data and error bars are mean ± SD (standard deviation) of three replicates.

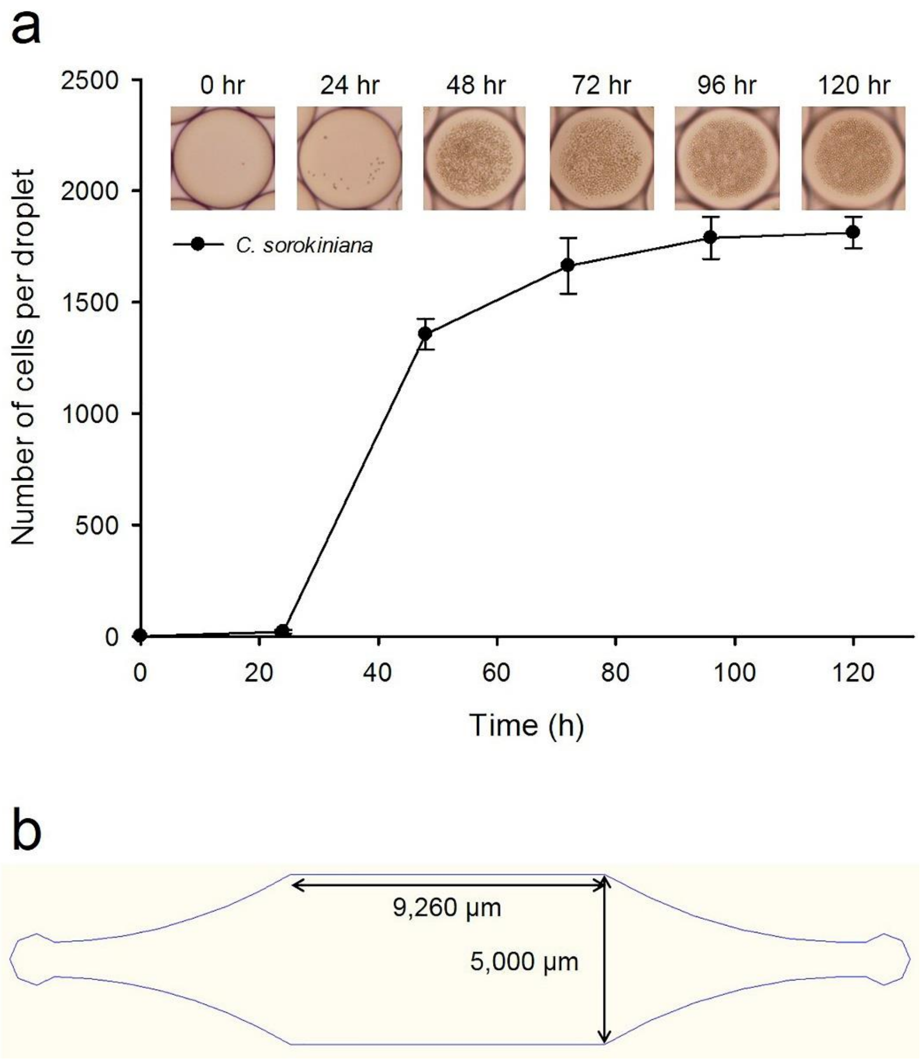

Supplementary Figure S4. Cultivation of microalgal cell in magnetic microdroplet. (a) Photoautotrophic growth kinetics of *Chlorella sorokiniana* in the microdroplet 5 mg mL^{-1} of dextran-coated superparamagnetic magnetite (Fe_3O_4) nanoparticles at 23 ℃ with 5% CO_2 under continuous light of 150 μmol photons m^{-2} s^{-1}. After 5 days of cultivation, the number of cells per droplets can be reached to about 2,000 (1.0×10^9 cells mL^{-1}) which is the same cell concentration of low-density droplet. (b) Design of microfluidic system for the photoautotrophic cultivation of microalgae. The height of microchamber is about 100 μm that can make the 160 μm sized droplets squashed resulting in the increase of contact area between the droplet and PDMS allowing the permeation of CO_2 increases[25].

Distance from the magnet (μm)	Fraction of empty droplets (%)	Fraction of high-density droplets (%)
420	3.49025974	
450	14.12337662	
480	75.78463203	
510	6.899350649	
540	2.597402597	
570		7.315759142
600		59.9822043
630		26.60644662
660		9.1433849

$$\mathrm{m} = \sum_{i=1}^{n} x_i p_i$$ (where, m: mean, x_i = random variable, p_i = probability)

•Empty droplet: 476.5340909 μm = 420 μm × 0.0349025974 + 450 μm × 0.1412337662 + 480 μm × 0.7578463203 + 510 μm × 0.06899350649 + 540 μm × 0.02597402597

•High-density droplet: 587.8601799 μm = 570 μm × 0.07315759142 + 600 μm × 0.599822043 + 630 μm × 0.2660644662 + 660 μm × 0.091433849

•The gap between empty and high-density droplets: 111.33 μm = 587.86 μm - 476.53 μm

Supplementary Table S1. To investigate the effects of various parameters on the amount of lateral displacement of the droplets, we set an observation region that was divided into 56 virtual parts at intervals of 30 μm at the end of the expansion channel (Fig. 2d). Under the optimum separation conditions (Flow rate: 820.14 μm s^{-1}, Magnet Mid, *y*-axis distance of magnet: 1205.61 μm, Droplet diameter: 160 μm), the empty and high-density droplets pass through the points where 476.53 μm and 587.86 μm away from the magnet, respectively. Therefore, the maximum gap between the empty and high-density droplets can be calculated as the 111.33 μm.

Supplementary Video S1. Separation of three different magnetic microdroplets (empty, low-density, high-density). Empty, low-, and high-density droplets flow into outlets 1, 2, and 3, respectively.
